# Inhibition of nuclear factor of activated T cells (NFAT) c3 activation attenuates acute lung injury and pulmonary edema in murine models of sepsis

**DOI:** 10.18632/oncotarget.24320

**Published:** 2018-01-25

**Authors:** Manjula Karpurapu, Yong Gyu Lee, Ziqing Qian, Jin Wen, Megan N. Ballinger, Luiza Rusu, Sangwoon Chung, Jing Deng, Feng Qian, Brenda F. Reader, Teja Srinivas Nirujogi, Gye Young Park, Dehua Pei, John W. Christman

**Affiliations:** ^1^ Pulmonary, Allergy, Critical Care and Sleep Medicine, Ohio State University Wexner Medical Center, Davis Heart and Lung Research Institute, Columbus, OH 43210, USA; ^2^ Department of Chemistry and Biochemistry, The Ohio State University, Columbus, OH 43210, USA; ^3^ Pulmonary, Allergy, Critical Care and Sleep Medicine, University of Illinois, Chicago, IL 60612, USA

**Keywords:** macrophage, NFAT’s, acute lung injury, pulmonary edema, CP9-ZIZIT

## Abstract

Specific therapies targeting cellular and molecular events of sepsis induced Acute Lung Injury (ALI) pathogenesis are lacking. We have reported a pivotal role for Nuclear Factors of Activated T cells (NFATc3) in regulating macrophage phenotype during sepsis induced ALI and subsequent studies demonstrate that NFATc3 transcriptionally regulates macrophage CCR2 and TNFα gene expression. Mouse pulmonary microvascular endothelial cell monolayer maintained a tighter barrier function when co-cultured with LPS stimulated NFATc3 deficient macrophages whereas wild type macrophages caused leaky monolayer barrier. More importantly, NFATc3 deficient mice showed decreased neutrophilic lung inflammation, improved alveolar capillary barrier function, arterial oxygen saturation and survival benefit in lethal CLP sepsis mouse models. In addition, survival of wild type mice subjected to the lethal CLP sepsis was not improved with broad-spectrum antibiotics, whereas the survival of NFATc3 deficient mice was improved to 40–60% when treated with imipenem. Passive adoptive transfer of NFATc3 deficient macrophages conferred protection against LPS induced ALI in wild type mice. Furthermore, CP9-ZIZIT, a highly potent, cell-permeable peptide inhibitor of Calcineurin inhibited NFATc3 activation. CP9-ZIZIT effectively reduced sepsis induced inflammatory cytokines and pulmonary edema in mice. Thus, this study demonstrates that inhibition of NFATc3 activation by CP9-ZIZIT provides a potential therapeutic option for attenuating sepsis induced ALI/pulmonary edema.

## INTRODUCTION

Alveolar macrophages are key innate immune cells that orchestrate the lung immune response. Studies from our laboratory and others have shown that administration of clodronate liposomes, which induced depletion of macrophages, suppressed endotoxin-induced neutrophilic inflammation in mouse lungs [1–3]. We have also shown that mice whose macrophages are PU.1-deficient, and thus functionally incompetent, have decreased lung inflammation and pulmonary edema in the setting of severe sepsis indicating a central role of macrophages in pulmonary inflammation and edema development [4]. However, it is unknown whether and how macrophages directly influence lung vascular permeability function that results in pulmonary edema. Our previous studies have identified critical protein factors in macrophages that play a role in initiation, mediation, and resolution of acute lung injury [4, 5]. We have examined the gene targets of sepsis in macrophages and discovered that NFATc3 is activated during sepsis and regulates expression of induced Nitric Oxide Synthase [6]. Our current studies indicated that NFATc3 also regulates CCR2, TNFα and few other inflammatory genes in macrophages upon LPS stimulation. Initially, NFATs were reported to regulate T-cell development, activation and differentially affect cellular function in cancer [7, 8]. NFATc3 activation and its functional significance in sepsis-induced ALI have not been examined in macrophages that are critical innate immune cells involved in ALI pathogenesis.

The first member of NFAT family, NFATc2 was discovered in T-cells as an inducible nuclear factor that binds to the antigen receptor response element-2 of the interleukin-2 promoter in human T cells [9, 10]. Subsequently, it was found that NFATs are not expressed exclusively in T cells, but are ubiquitously expressed in various immune and non-immune cells in the vertebrate systems [11–18]. NFATs are critical regulators of developmental control functions including cell fate determination, embryonic development, and organogenesis of the cardiac, hematopoietic, skeletal, and neuronal systems [19–22]. The NFAT protein family consists of five members referred as NFAT1 (NFATc2 or NFATp), NFAT2 (NFATc1 or NFATc), NFAT3 (NFATc4), NFAT4 (NFATc3 or NFATx), and NFAT5 (tonicity-responsive enhancer-binding protein (TonEBP) [23]. NFAT1, NFAT2, NFAT3 and NFAT4 are activated by cellular calcium influx and NFAT5 is activated by osmotic stress. The calcium-responsive NFAT isoforms (NFAT1-NFAT4) exist in hyper-phosphorylated states within the cytoplasm and are activated by increased intracellular calcium levels via de-phosphorylation by calcineurin and nuclear translocation [24]. NFATs that translocate to the nucleus activate transcription of downstream target genes, thus directly linking calcium signaling to gene expression. To date, only calcineurin is known to activate the NFAT1-4 transcription factors thereby controlling the expression of a broad range of genes. NFAT proteins were implicated in pathogenesis of various inflammatory pathologies. Research to identify NFAT inhibitors is underway since their discovery but yielded little success [25, 26].

Aramburu *et al.*, have discovered a 14mer VIVIT peptide with higher specificity that inhibits NFAT calcineurin interaction thereby inhibiting dephosphorylation/nuclear translocation of NFAT1-4 [27]. VIVIT has a modest binding affinity for calcineurin (*K*_D_ = 500 nM). To improve its potency, the two valine residues in VIVIT were replaced with tert-leucine and the resulting peptide, ZIZIT, is an order of magnitude more potent (*K*_D_ = 43 nM) [28]. In this work, we covalently attached ZIZIT to a cyclic cell-penetrating peptide to generate a highly potent and cell-permeable calcineurin inhibitor, CP9-ZIZIT [29, 30]. Compared to VIVIT, CP9-ZIZIT is expected to possess higher affinity for calcineurin, better cell permeability, and improved stability against proteolysis which would predict higher efficacy in inactivating NFATs [28, 30].

Knockout mice of each NFAT family member exhibit distinct phenotypic defects, possibly due to the cell type-specific regulation and expression profiles of each NFAT. However, *in vivo,* the contribution of NFATs to the macrophage-mediated pulmonary innate immune system response during sepsis-induced ALI has not been addressed. In our earlier studies, we reported that LPS activated NFATc3 regulates macrophage-specific iNOS, which is critical for macrophage bactericidal activity and their role in host defense [6]. In the current manuscript, we have identified a novel role for NFATc3 in the regulation of inflammatory genes produced by macrophages during murine sepsis-induced ALI, and inhibiting NFATc3 activation with the high affinity CP9-ZIZIT peptide notably attenuated pulmonary edema and lung wet to dry weight ratios during LPS inhalation-induced ALI in mice.

## RESULTS

### LPS activates NFATc3 selectively in macrophages and NFATC3 is a transcriptional regulator of inflammatory genes

**Figure 1:**
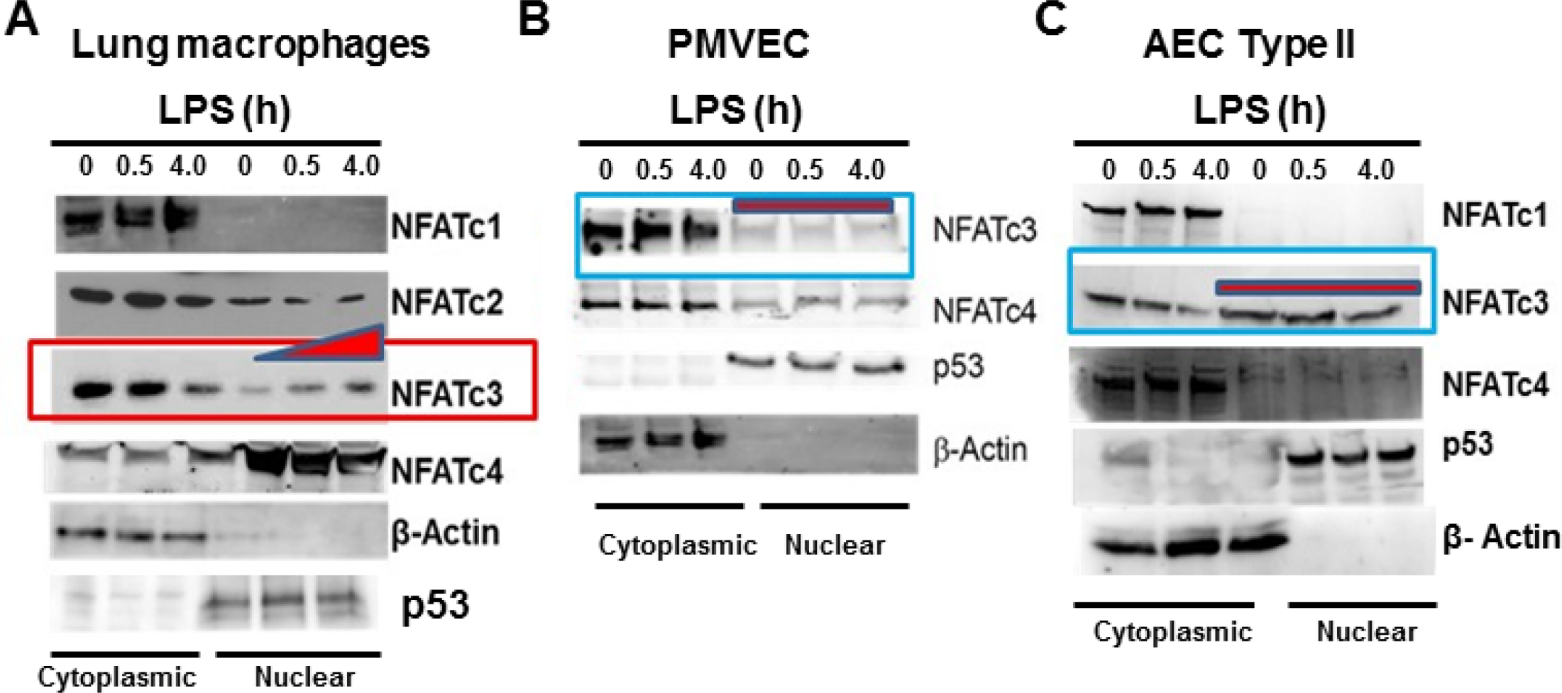
LPS induced NFATc3 activation and translocation is macrophage specific (**A**) Mouse total lung macrophages were stimulated with LPS (100 ng/mL for 0.5 and 4.0 h). The cytoplasmic and nuclear proteins were isolated using Peirce Nuclear and Cytoplasmic protein isolation kit and equal amount of proteins were immunoblotted for NFATc1, NFATc2, NFATc3 and NFATc4 to determine their cytoplasmic vs. nuclear distribution. (**B**) Mouse PMVEC were treated with LPS (100 ng/mL for 0.5 and 4.0 h). The cytoplasmic and nuclear proteins were isolated and analyzed for NFAT1-NFAT4 nuclear translocation. (**C**) Mouse AEC type II cells were treated with LPS (100 ng/mL for 0.5 and 4.0 h) and analyzed for NFAT1-NFAT4 nuclear translocation. Purity of cytoplasmic and nuclear proteins is determined by immunoblotting with β-Actin and p53.

We observed that *in vitro* treatment with LPS, an inducer of sepsis-like pathophysiology in mice, results in induction of NFATc3 activation in macrophages. Cytoplasmic and nuclear proteins from LPS-stimulated total lung macrophages were immuno-blotted with antibodies specific to NFAT1-NFAT4. LPS induced a highly specific, time-dependent translocation of NFATc3 from the cytoplasm to the nuclear compartment (Figure 1A). NFATc1 and NFATc4 are constitutively present in the cytoplasm and nucleus, respectively. NFATc2 was present predominantly in cytoplasm indicating that only NFATc3, among the tested NFATs, undergoes activation/nuclear translocation in response to LPS treatment in lung macrophages. Although, NFATc3 is abundantly expressed in mouse alveolar epithelial type II cells (AEC Type II), LPS stimulation does not result in NFATc3 translocation from cytoplasm to nucleus similar to pulmonary microvascular endothelial cells (PMVECs) (Figure 1B–1C). Thus, compared to pulmonary microvascular endothelium and alveolar type II epithelium, NFATc3 alone is activated by LPS in lung macrophages (Figure 1A–1C).

**Figure 2:**
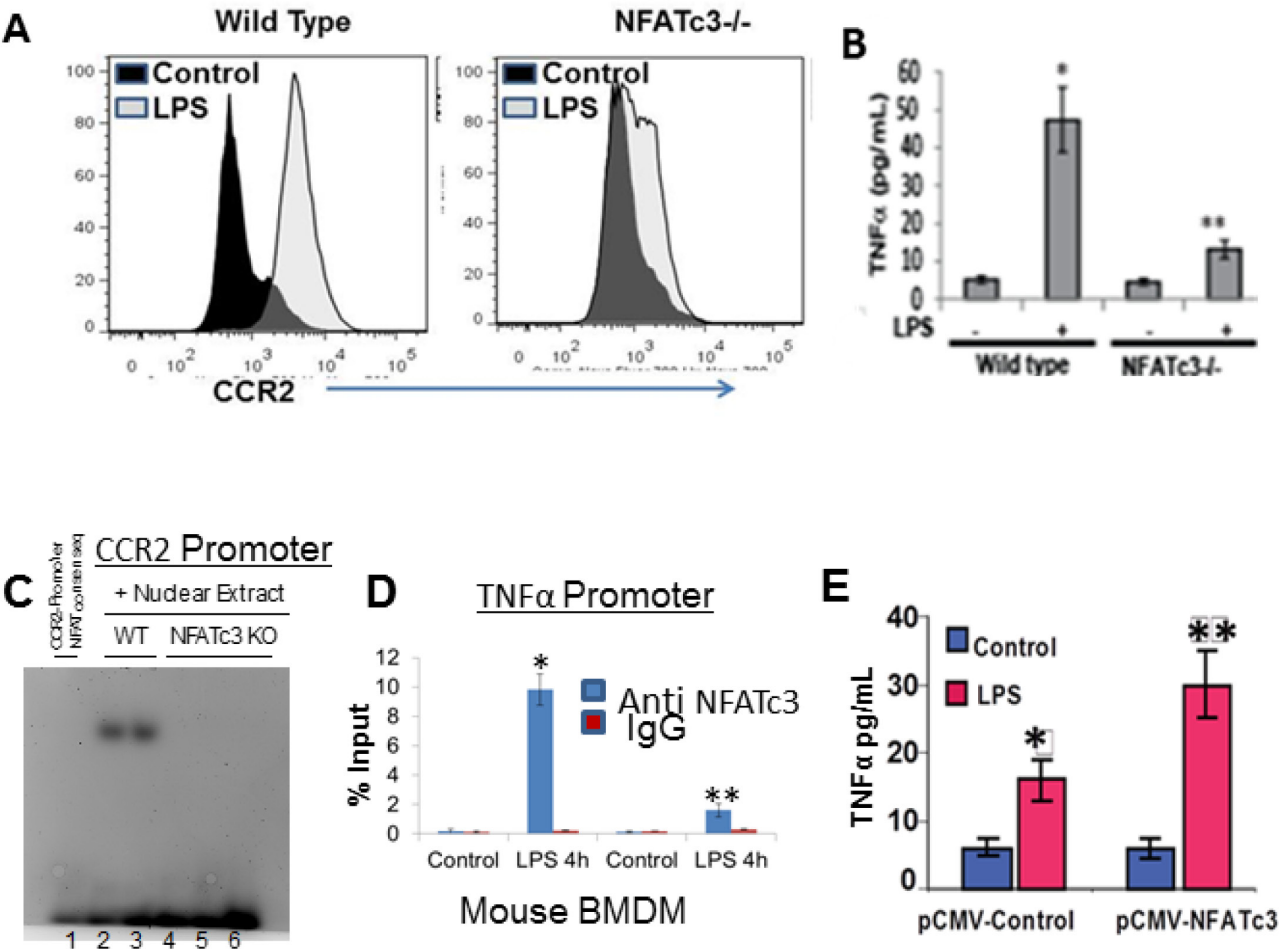
NFATc3 transcriptionally regulates CCR2 and TNFα expression BMDMs from WT and NFATc3^−/−^ mice were stimulated with LPS for 24 h and analyzed for (**A**). Expression of CCR2 by flow cytometry (**B**) Extracellular medium for TNFα release by ELISA. (**C**) Binding of NAFTc3 to the CCR2 promoter was determined by Electrophoretic mobility shift assay. (**D**) Binding of NAFTc3 to the TNFα promoter was determined by Chromatin ImmunoPrecipitation using QIAGEN One day ChIP kit. (**E**) NFATc3 was overexpressed in NFATc3^−/−^ BMDMs by electroporation with a pCMV-NFATc3 plasmid and stimulated with LPS for 24 h. LPS induced TNFα release in to the culture medium was measured using R&D systems Quantikine TNFα ELISA kit. B-D-E, ^*^P ≤ 0.01 LPS vs. control; ^**^p ≤ 0.001 NFATc3^−/−^ vs. WT or pCMV-NFATc3 vs pCMV-Control.

Since NFATc3 is selectively activated in lung macrophages by LPS, we next aimed to determine the role of activated NFATc3 on bone marrow derived macrophage (BMDM) inflammatory gene expression by using a PCR array for mouse inflammatory and autoimmunity genes (SABioscience, PAMM-077A). WT macrophages, stimulated with LPS showed distinct upregulation of inflammatory genes such as TNFα, iNOS, CCR2, and CCL2 (Supplementary Table 1). Comparison of LPS stimulated NFATc3^−/−^ and WT BMDM gene expression indicated distinct down regulation of LPS induced CCR2 and TNFα in NFATc3^−/−^ macrophages (Figure 2A–2B), suggesting that NFATc3 regulates macrophage gene expression thereby regulates pathogenesis of sepsis induced-ALI. To test this hypothesis, we characterized NFATc3 deficient and WT macrophages and measured the outcomes of sepsis-induced ALI in NFATc3^−/−^ and WT mice. Next, we determined if NFATc3 transcriptionally regulates CCR2 and TNFα by binding to their promoters. LPS increased NFATc3 binding to NFAT consensus sequence in CCR2 and TNFα and promoters in WT BMDM whereas there was no such increase in NFATc3^−/−^ BMDM (Figure 2C–2D). In addition, TNFα released into the media after LPS stimulation in NFATc3^−/−^ BMDMs was restored to levels comparable with those of WT BMDMs, when NFATc3^−/−^ cells were electroporated with NFATc3 expressing plasmid and stimulated with LPS (Figure 2E). NFATc3 overexpression in NFATc3^−/−^ BMDMs confirmed the functional role of NFATc3 in regulation of TNFα.

### NFATc3^−/−^ macrophages modulate PMVEC monolayer barrier function

**Figure 3:**
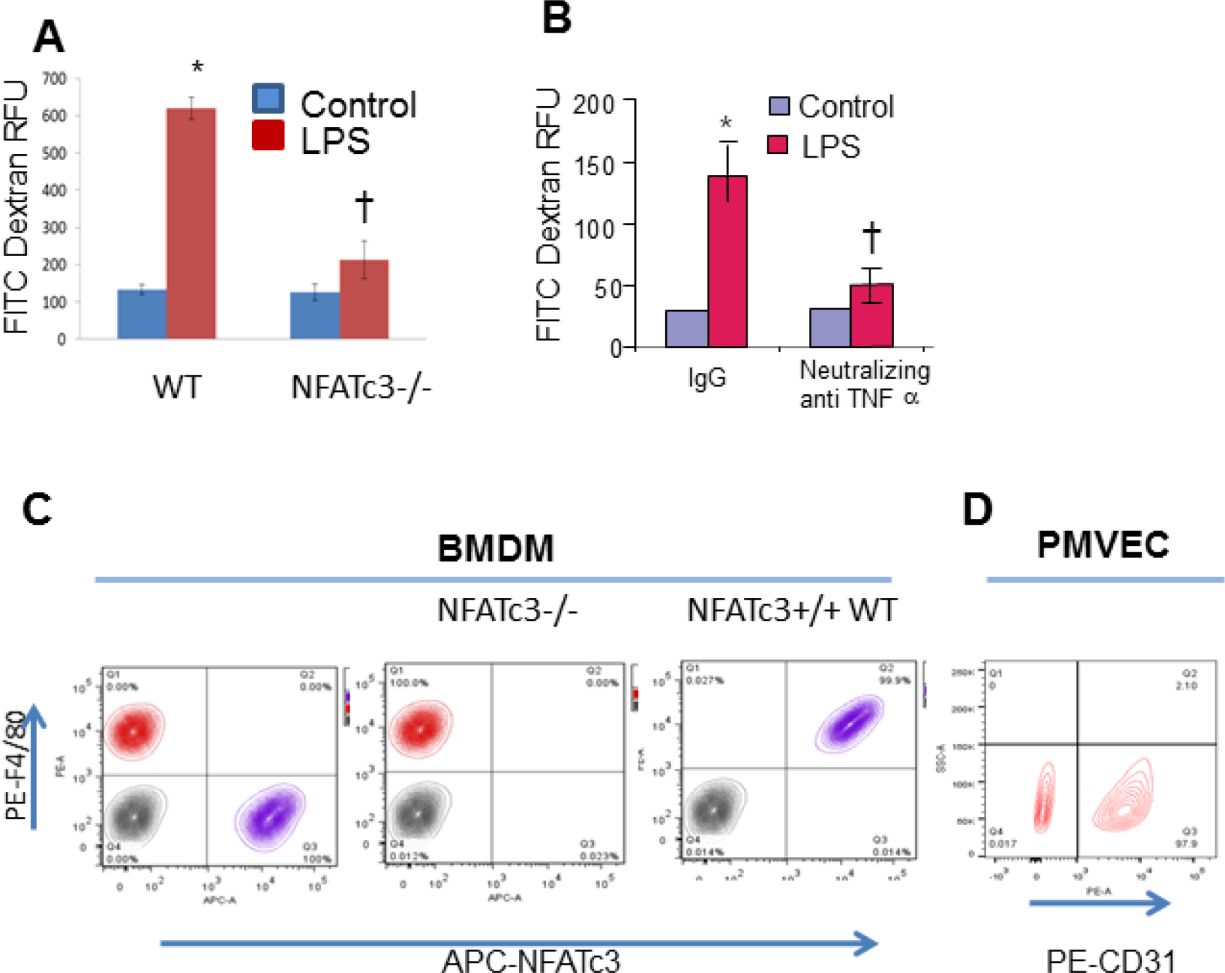
NFATc3^−/−^ macrophages positively modulate mouse PMVEC permeability Mouse BMDM stimulated with LPS (100 ng/mL) for 24 h were co-cultured with mouse PMVEC monolayer grown on cell culture inserts, added with FITC-Dextran to the trans wells at the time of initiating co-culture. (**A**) Flux of FITC-Dextran in WT and NFATc3^−/−^ macrophage co-culture was determined by measuring fluorescence of the medium in the bottom wells as described in methods. Similarly, (**B**) FITC-Dextran flux in neutralizing TNFα pre- treated WT BMDM cells is quantitated as described in methods. (**C**) Homogeneity of NFATc3^−/−^ & WT BMDM was determined by staining with anti-APC-NFATc3 and F480. (**D**) Homogeneity of PMVEC is confirmed by staining with anti-PE-CD31. A–B ^*^p ≤ 0.001 LPS vs PBS; ^†^*p* ≤ 0.01 NFATc3^−/−^/NeutralizingTNFα vs NFATc3^+/+^(WT)/Normal IgG.

NFATc3^−/−^ macrophages distinctly express decreased inflammatory molecules indicating a potential link between NFATc3-deficient macrophages and integrity of surrounding microvascular monolayer barrier properties. Therefore, we determined whether NFATc3 deficiency in macrophages would affect the mouse PMVEC monolayer barrier function. To test this, we co-cultured macrophages with PMVECs and determined the PMVEC monolayer barrier function. Hanging transwell cell culture inserts were seeded to 100% confluence with mouse PMVEC isolated by CD31 Miltenyi magnetic beads and further co-cultured with NFATc3^−/−^ or WT BMDMs that were grown in 24 well plates stimulated with LPS for 8 hrs. At the time of initiation of co-culture, FITC-Dextran (10 kDa) was added to PMVEC monolayer grown cell culture inserts and flux of FITC-Dextran across the monolayer was measured after 24 h. PMVEC cell culture inserts placed on control BMDMs (PBS) showed no detectable levels of FITC-dextran in the bottom wells indicating maintenance of tighter PMVEC monolayer barrier function whereas inserts placed into wells with LPS treated WT BMDMs showed increased accumulation of FITC-dextran in bottom wells (Figure 3A). Interestingly, PMVEC co-cultured with LPS stimulated NFATc3^−/−^ BMDMs showed decreased FITC-dextran flux across the monolayer compared to WT BMDM (Figure 3A). Homogeneity of NFATc3^−/−^ BMDMs was confirmed by staining for the macrophage marker F4/80 (Ab F4/80-PE) and NFATc3 (anti-NFATc3-APC, Figure 3C), and PMVECs were confirmed by CD31-PE staining (Figure 3D). Effect of NFATc3 driven macrophage TNFα expression on PMVEC monolayer permeability was determined using neutralizing TNFα antibody in co-cultures. Pretreatment of WT-BMDM with neutralizing TNFα antibody significantly decreased FITC-dextran flux, indicating reduced permeability of mouse PMVEC monolayer (Figure 3B). These data established a strong correlation between macrophage NFATc3 dependent TNFα expression and pulmonary microvascular endothelial cell barrier function.

### NFATc3^−/−^ mice compared to WT mice show healthy lung parameters during sepsis-induced ALI

**Figure 4:**
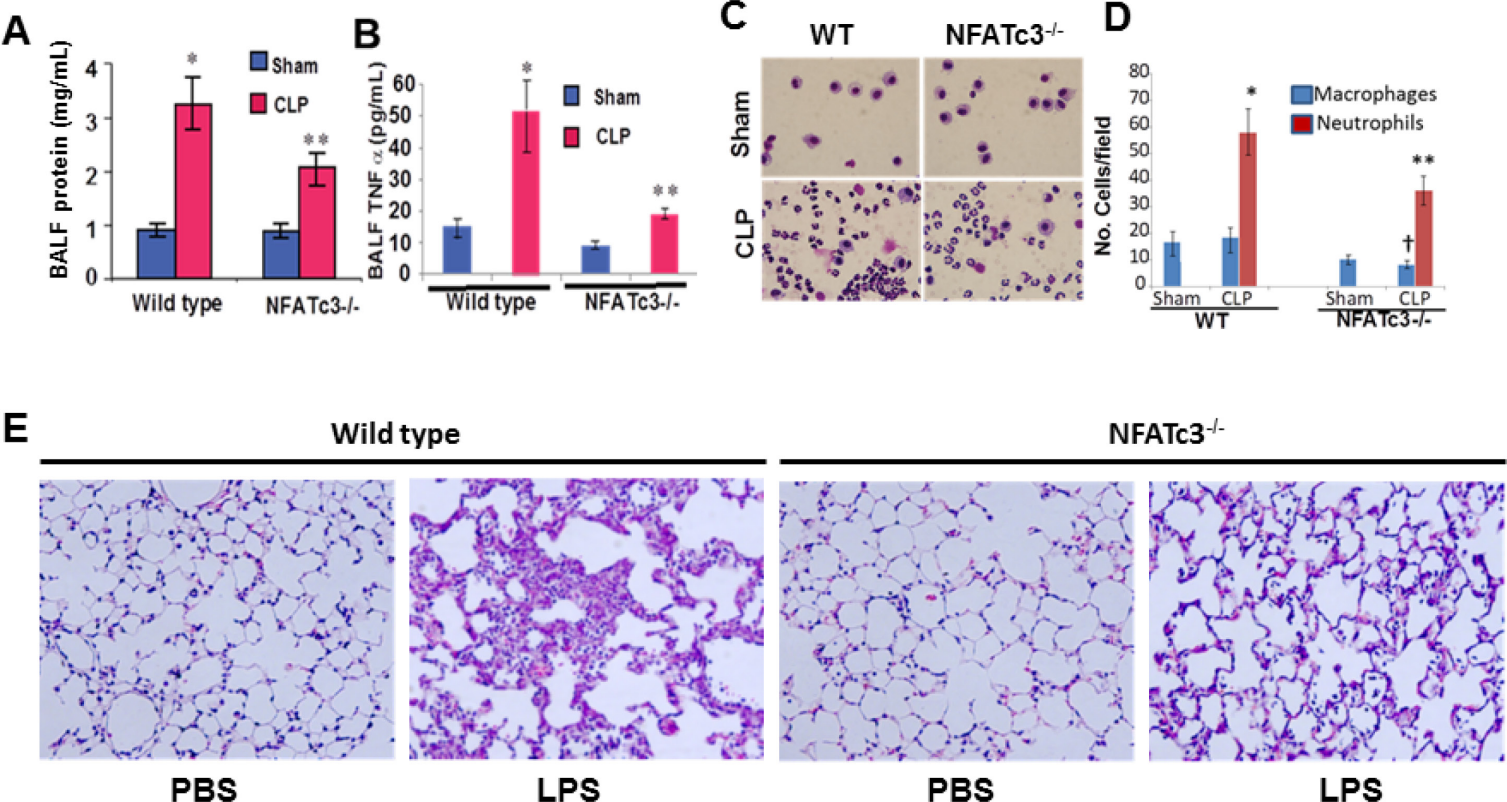
NFATc3^−/−^ mice subjected to abdominal sepsis by CLP show healthy lung parameters NFATc3^−/−^ and WT mice were subjected to CLP or sham surgery and analyzed after 24 h to determine (**A**) protein leakage in to BALF, (**B)**. TNFα released in to BALF was measured by ELISA (**C–D**) Hema 3.0 Stained Neutrophils and macrophages in BALF. (**E**) NFATc3^−/−^ and WT mice were subjected to LPS (i.p 15 mg/kg) sepsis for 16 h. Lungs were fixed, embedded in paraffin, sectioned and H&E stained. Lung histology showing neutrophilic infiltration in to NFATc3^−/−^ and wild type mice. (A–D) ^*^p ≤ 0.001 WT-CLP vs WT- Sham; ^**^p ≤ 0.01 NFATc3^−/−^ CLP vs WT-CLP; ^†^*p* ≤ 0.05 macrophages in NFATc3^−/−^ CLP vs WT-CLP.

**Figure 5:**
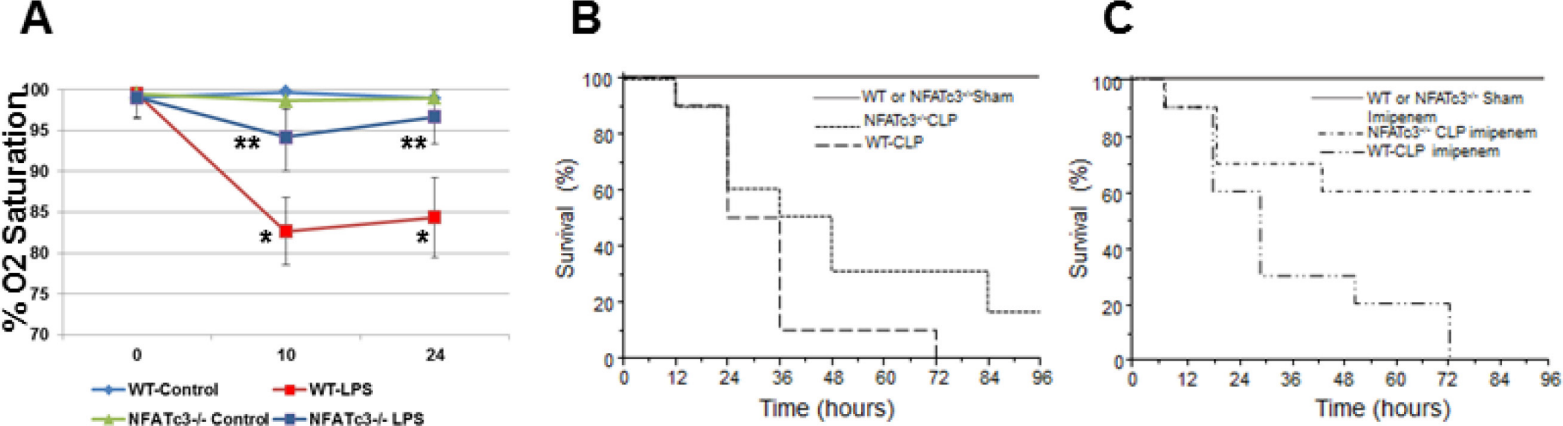
NFATc3^−/−^ mice show improved arterial Oxygen saturation and survival compared to wild type mice (**A**) NFATc3^−/−^ and wild type mice (*N* = 5) were injected with 15 mg/kg LPS i.p and the arterial oxygen saturation was measured using STARR Life Sciences Corp. Pulse Oximeter. (**B**–**C**) NFATc3^−/−^ and wild type mice (*N* = 5 sham control group; *N* = 10 CLP group) were subjected to severe CLP, maintained on regular or imipenem monohydrate supplemented water and the survival was monitored over a period of 96 h. A^*^ *p* ≤ 0.001 WT-LPS vs. WT-Control; ^**^ *p* ≤ 0.01 NFATc3^−/−^ LPS vs. WT-LPS.

**Figure 6:**
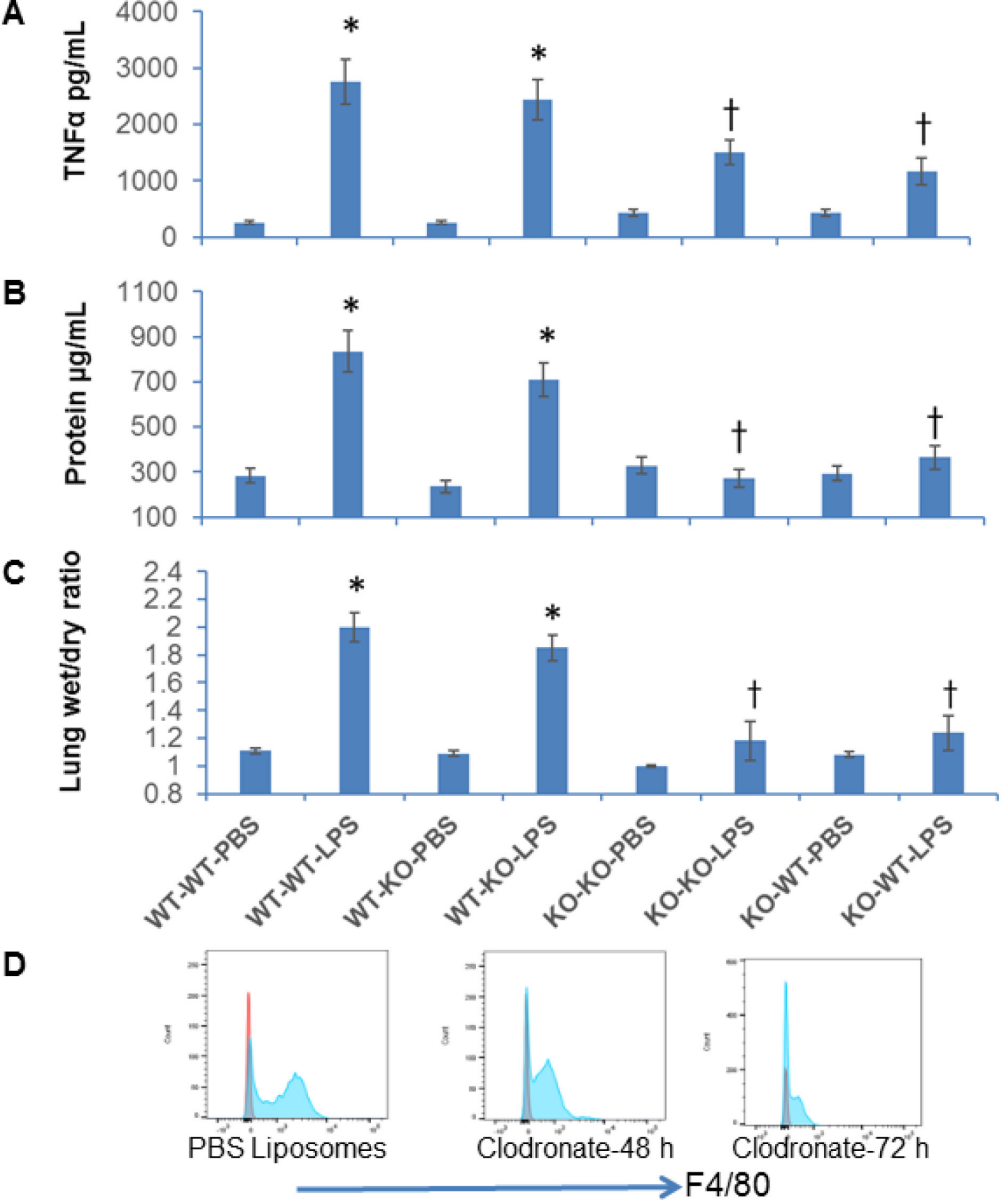
Adoptive transfer of NFATc3^−/−^ macrophages protects mice against LPS induced acute lung injury WT or NFATc3^−/−^ mice were pretreated with codronate liposomes for 72 h to deplete lung resident macrophages. The macrophage deleted mice were adoptively transferred with lung macrophages from reciprocal NFATc3 background and administered with i.p LPS (15 mg/kg). Pulmonary inflammation and edema was measured by (**A**) TNFα in BALF (**B**) BALF protein content (**C**) lung wet to dry ratios. (**D**) Depletion of lung resident macrophage population after clodronate liposome administration compared to PBS-liposomes was determined by flow sorting cells for CD45 and CD11b/F4/80 positive population. ^*^p ≤ 0.01 WT-WT or WT-KO/LPS vs WT-WT or WT-KO/PBS; ^†^*p* ≤ 0.01 KO-KO-LPS or KO-WT-LPS vs WT-WT-LPS or WT-KO-LPS.

Since macrophages with altered expression levels of TNFα, CCR2 and iNOS are expected to affect lung inflammation, homeostasis and host resistance, we determined the functional implication of NFATc3 deletion on sepsis-induced ALI in mouse CLP sepsis models. We subjected NFATc3^−/−^ and WT littermates to moderate CLP or sham surgery. At 24 hours, the degree of pulmonary edema and the intensity of inflammation were assessed by measuring protein and TNFα levels in broncho alveolar lavage fluid (BALF) (Figure 4A–4B). WT mice subjected to moderate CLP showed greater protein leakage, TNFα expression, and neutrophil numbers in BALF compared to NFATc3^−/−^ mice (Figure 4A–4D). These data suggested that NFATc3-deficient mice are protected from ALI associated pulmonary edema during polymicrobial abdominal sepsis. However, in our previous study, NFATc3^−/−^ polymicrobial septic mice have been found to have increased bacterial colony forming units (CFUs) per gram of lung tissue [6]. It is interesting to note that in our study the neutrophilic infiltration in NFATc3^−/−^ mouse lung was marginally reduced to only about 45% of WT, which is apparently a level of neutrophilic lung inflammation that is adequate for bacterial phagocytosis but may not be sufficient to cause permeability pulmonary edema (Figure 4C–4D). Furthermore, the macrophage numbers in BALF were also significantly reduced in NFATc3^−/−^ mice (Figure 4C–4D). Similarly, NFATc3^−/−^ mice when challenged with LPS (15 mg/kg i.p) showed decreased neutrophilic inflammation and intact alveolar architecture compared to wild type mice (Figure 4E). Next, we measured the pulmonary arterial oxygenation using MouseOx Pulse Oximeter (STARR Life Sciences Corp). As shown in Figure 5A, NFATc3^−/−^ mice maintained 9–12% increased arterial oxygenation at 8 h and 24 h post LPS (15 mg/kg i.p.) injection compared to WT mice. Our earlier studies supported NFATc3 activation induced iNOS expression to be necessary for maintaining antibacterial pulmonary host defense [6] whereas current results show that NFATc3 activation mediates alveolar capillary barrier dysfunction (Figures 3A, 4A, 5A). This raises concerns regarding the relative benefit of protecting alveolar capillary barrier function at the expense of a detrimental impact on antibacterial host defense by targeting NFATc3 activation. To address this important clinical concern, we investigated whether the increase in bacterial infection observed in NFATc3^−/−^ mice translated into a poorer prognosis of CLP-induced mortality or whether the improved arterial oxygenation and decreased pulmonary vascular leakage or edema more than compensated, leading to a better outcome. NFATc3^−/−^ and WT mice were subjected to severe sepsis by using a lethal model of CLP and were monitored for survival over 96 h. Compared to WT the NFATc3^−/−^ mice showed a 20% improvement in survival rate at 96 h whereas, WT-CLP mice showed 100% lethality by 72 hours (Figure 5B). Furthermore, 60% of NFATc3-deficient mice survived with the administration of the broad-spectrum antibiotic imipenem (Figure 5C). In contrast, the lethality of the WT severe CLP mice was not improved any further by Imipenem (Figure 5C). These data, all together, indicate that NFATc3^−/−^ mice show decreased pulmonary protein leakage, neutrophilic infiltration, improved arterial oxygenation and survival despite the excessive bacterial load detected in lung tissue in our previous study [6]. Despite the compromised host defense in NFATC3^−/−^ mice, management with broad spectrum antibiotics improved the overall lung function during sepsis induced ALI and survival. To determine if the decreased pulmonary edema in NFATc3^−/−^ mice is due to macrophage mediated phenotype, WT and NFATc3^−/−^ mice are depleted of lung macrophages by clodronate liposomes for 72 h and adoptively transferred with lung macrophages from reciprocal genetic back ground as described earlier [5]. The recipient mice are subjected to LPS or PBS (i.p) challenge. After 24 h, WT mice that received NFATc3^−/−^ lung macrophages and challenged with LPS showed decreased lung wet/dry ratio and BALF protein/TNFα (Figure 6A–6C). NFATc3^−/−^ mice tLPS showed increased lung wet/dry ratio, BALF protein and TNFα similar to WT mice (Figure 6A–6C). As shown in Figure 6D, Clodronate liposomes depleted resident lung macrophages in recipient mice from 86% to 34%. Lung tissue homogenates from NFATc3^−/−^ mice showed no expression of NFATc3 and in addition no compensatory expression of NFATc1, NFATc2, and NFATc4 was observed (Supplementary Figure 3).

### NFATc3-Calcineurin inhibitors offer protective effect against sepsis-induced ALI and pulmonary edema

**Figure 7:**
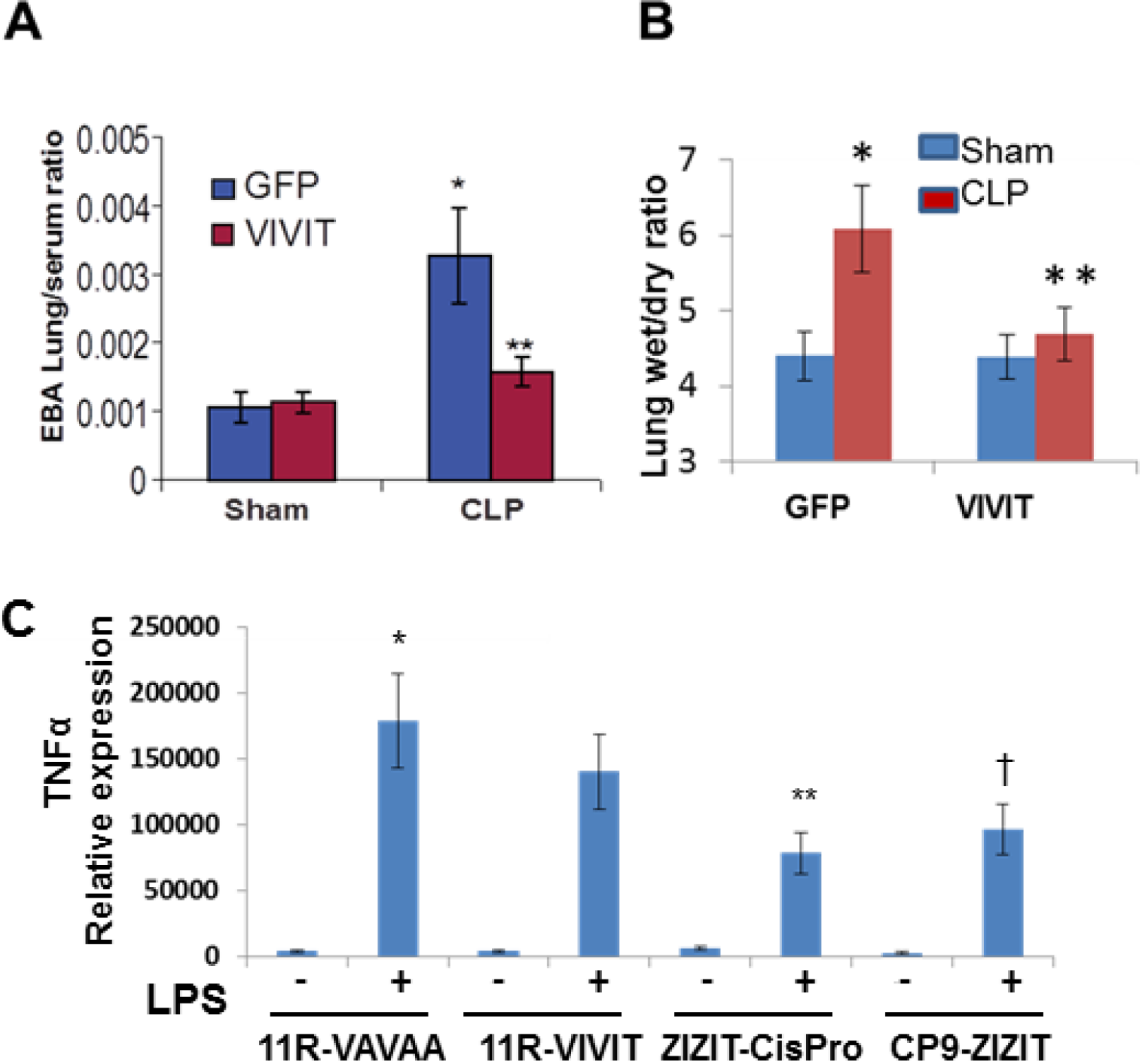
Pharmacological inhibition of NFAT attenuates pulmonary edema and inflammatory cytokines during sepsis-induced ALI Wild type mice were administered with GFP-VIVIT or GFP plasmid containing liposomes 24 h before CLP or sham surgery. (**A**) 2% Evans Blue Dye was injected retro-orbitally in to Sham and CLP mice and after 30 minutes mice were euthanized and lung vascular permeability was quantified by EBA extravasation. (**B**) Pulmonary edema was determined by differences in lung wet to dry weight ratios. In (A–B), ^*^p ≤ 0.01 CLP vs Sham; or GFP-VIVIT vs GFP. (**C**) BMDM were pretreated with 1 μM 11R-VAVAA, 11R-VIVIT, ZIZITcisPro or CP9-ZIZIT, separately and challenged with PBS/LPS for 8 h. TNFα transcript levels were measured by reverse transcribing total Cdna and TNFα specific primers. ^*^p ≤ 0.01 LPS vs PBS; ^**^p ≤ 0.05 ZIZITcisPro/LPS vs. 11R-VIVIT/LPS. ^†^*p* ≤ 0.01 CP9-ZIZIT/LPS vs 11R-VIVIT/LPS.

**Figure 8:**
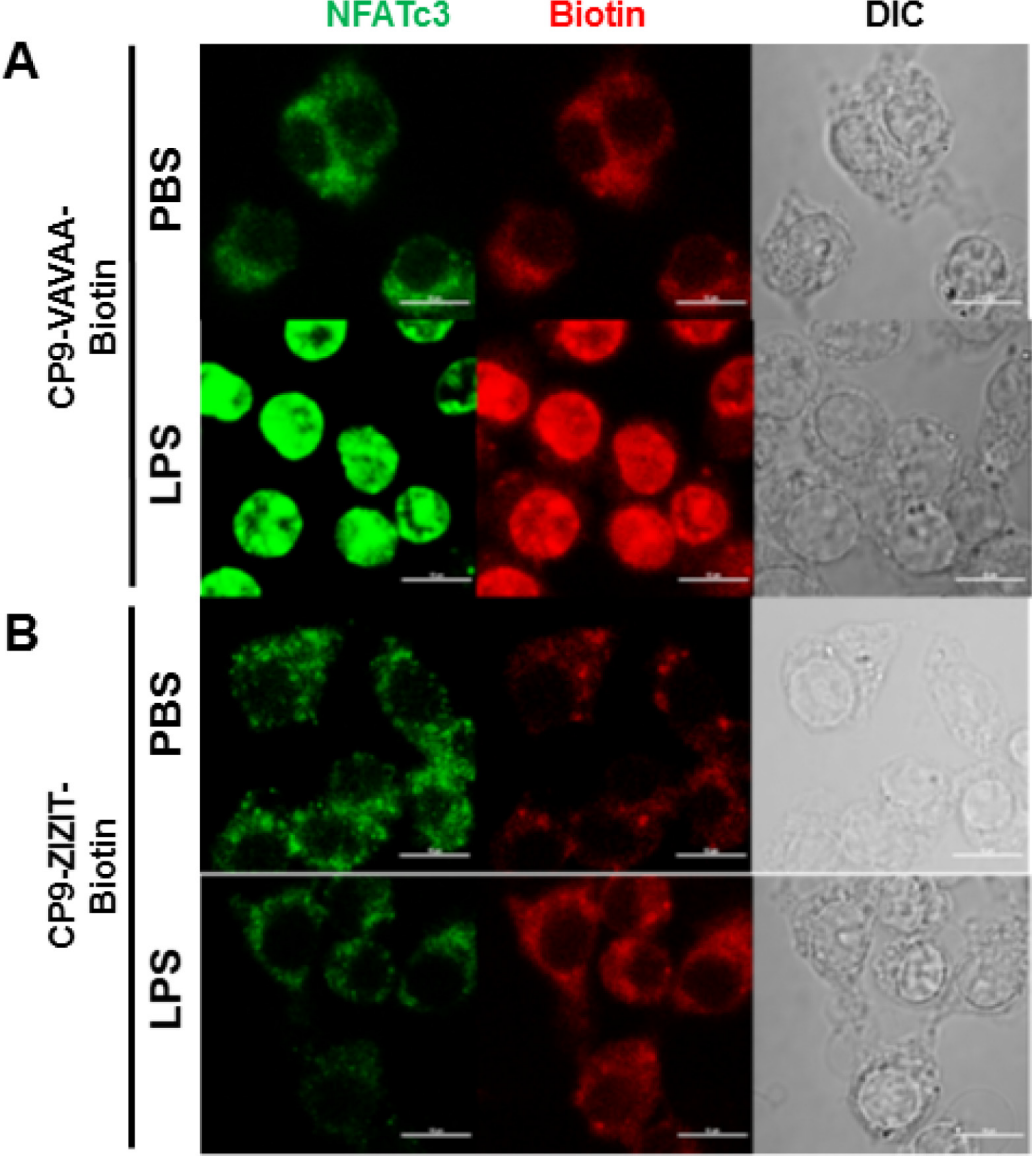
CP9-ZIZIT inhibits LPS induced NFATc3 nuclear translocation Mouse BMDM were pretreated with 1 μM of (**A**) control CP9-VAVAA or (**B**) CP9-ZIZIT inhibitor peptides and stimulated with PBS or LPS (100 ng/mL) for 1 hour. Localization of NFATc3 (green) and peptides (CP9-VAVAA or CP9-ZIZIT in red) with PBS/LPS stimulation is determined using Zeiss LSM 510 Meta microscope (Carl Zeiss MicroImaging, Inc.) equipped with 488 nm and 543 nm excitation lasers. Single confocal optical sections (pinhole set to achieve 1 Airy unit) are shown (*n* = 7–10 cells/group). Bars correspond to 10 μm.

**Figure 9:**
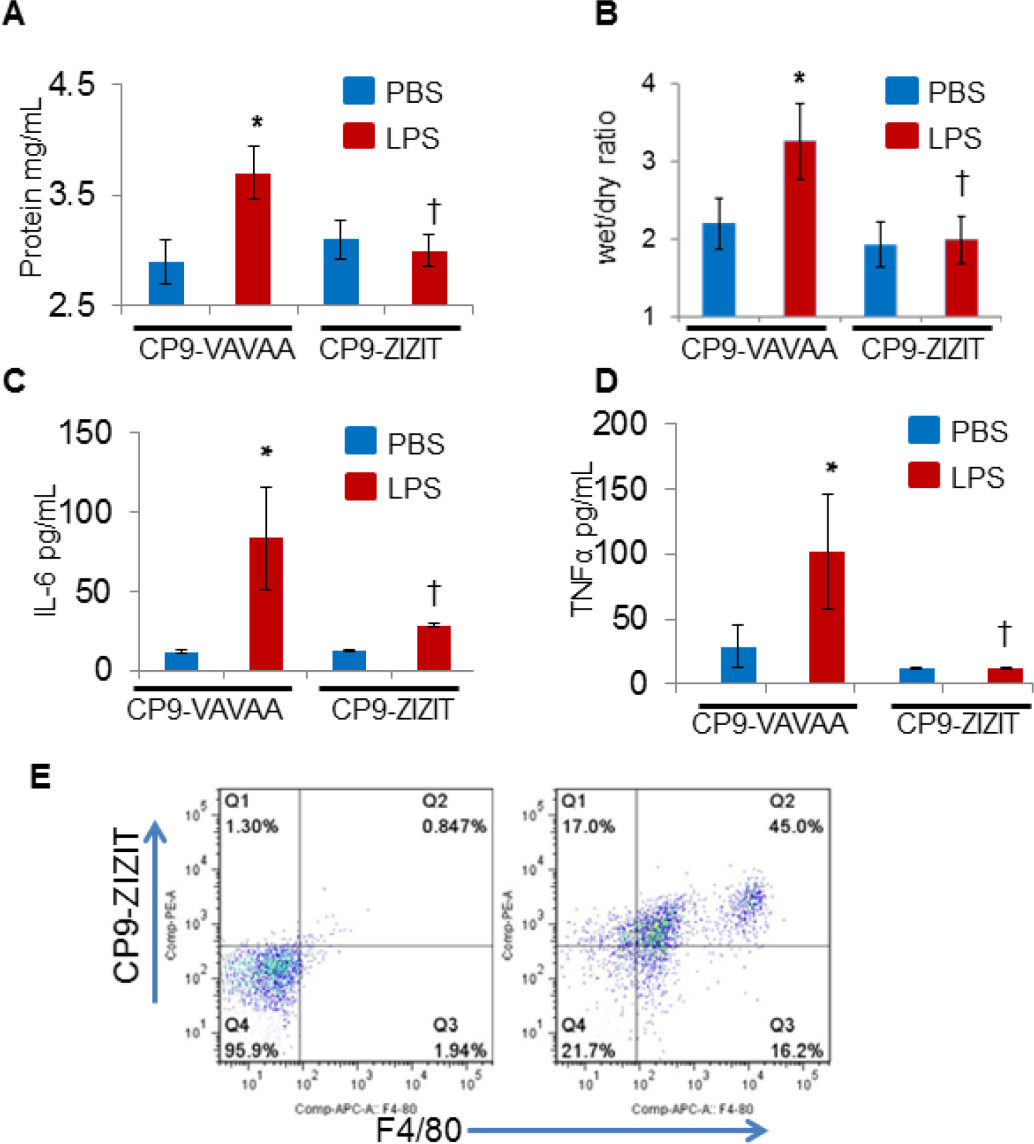
CP9-ZIZIT attenuates LPS induced pulmonary edema and cytokine storm in murine models of endotoxemia WT mice were administered with CP9-ZIZIT or CP9-VAVAA at 1 mg/kg dose 1 h before nasal insufflation of LPS (10 μg/kg). (**A**–**B**) Pulmonary edema was measured by total BALF protein content and lung wet to dry ratios. (**C**–**D**) IL6 and TNFα release in to BALF during sepsis-induced ALI was determined using R&D systems Quantikine IL6 and TNFα ELISA kits respectively. (**E**) Uptake of CP9-ZIZIT-Biotin by total lung macrophages is shown by flow cytometry. ^*^p ≤ 0.01 LPS vs PBS; ^†^*p* ≤ 0.01 CP9-ZIZIT vs CP9-VAVAA.

To confirm the *in vivo* results observed in the NFATc3-deficient mice, we next aimed to determine whether pharmacological inhibition of NFAT by a VIVIT expressing plasmid attenuates sepsis-induced ALI. GFP or GFP-VIVIT plasmids were delivered by intra-tracheal injection of plasmid-containing liposomes into WT mice. After 24 h, GFP control and GFP-VIVIT liposome injected mice were subjected to CLP or sham surgery and analyzed after 24 h for CLP-induced ALI and pulmonary edema. GFP-VIVIT plasmid transfected mice showed a significant decrease in both EBA extravasation and lung wet to dry ratio compared to GFP-control transfected mice (Figure 7A–7B) indicating that VIVIT expression is associated with improved alveolar endothelial barrier function. To this end, we conjugated a recently developed, highly potent, and proteolytically stable peptide inhibitor against calcineurin, ZIZIT, to a powerful cyclic cell-penetrating peptide [28, 29, 30]. The resulting peptide, CP9-ZIZIT, is a highly potent (*K*_D_ = 43 nM) and cell-permeable calcineurin-NFAT interaction inhibitor. CP9-ZIZIT peptide (Supplementary Figures 1, 2) used at much lower concentration (1 μM) attenuated TNFα gene expression in LPS stimulated BMDMs (Figure 7C). CP9-ZIZIT clearly inhibited LPS induced nuclear translocation of NFATc3 as evidenced by immunofluorescence staining (Figure 8A, 8B). CP9-ZIZIT-Biotin/CP9-VAVAA-Biotin peptides were detected with anti-biotin-Streptavidin-PE and NFATc3 with antiNFATc3-antiRabbit-FITC (Figure 8A, 8B). More importantly, *in vivo*, CP9-ZIZIT pretreated mice (1 mg/kg body weight, intranasal delivery) subjected to ALI by intranasal LPS instillation developed attenuated pulmonary edema and decreased cytokines (Figure 9A–9D). Similar to NFATc3^−/−^ mice, CP9-ZIZIT pretreated mice showed decreased pulmonary edema, BALF cytokines and lung wet to dry ratios during sepsis-induced ALI. The uptake of CP9-ZIZIT-biotin/CP9-VAVAA-biotin was around 45–50% in lung macrophages (Figure 9E).

## DISCUSSION

Innate immunity in response to pathogens relies on evolutionary conserved signaling pathways, involving the NF-κB transcription factor. However, recent literature suggests that the nuclear factor of activated T-cell transcription factors, that share ‘Rel homology’ DNA binding domain similar to NF-κB also contribute to innate immune response regulation. Exposure to microbial pathogens or inflammatory stimuli induces the activation of NFAT's in innate immune cells, including dendritic cells, granulocytes, macrophages and mast cells [11, 12, 31, 32]. We reported earlier that activated NAFTc3 in macrophages regulates iNOS expression and thereby host microbial defense in murine abdominal sepsis models [6]. An immediate study by Zhang *et al.*, (2014) implicated NFATc3 activation in regulating neutrophil recruitment, systemic inflammation and T-cell dysfunction during abdominal sepsis [33]. Current study using NFATC3^−/−^ mice lead to an important finding that NFATc3 plays a pivotal role in the pathogenesis of sepsis induced ALI and pulmonary edema in murine models of sepsis.

From our findings, NFATc3 activation during sepsis conditions appears to be specific for macrophages and was not observed in lung structural cells. Interestingly, NFATc3-deficient macrophages when stimulated with LPS showed attenuated expression of several cytokines, chemokines, and their receptors, thus translating to a profound phenotypic effect of NFATc3 deletion in sepsis induced ALI. NFATc3 deletion showed decreased pulmonary edema, neutrophilic inflammation, improved arterial oxygenation and survival during CLP-induced sepsis in mice. Binding of NFATc3 to CCR2, iNOS and TNFα promoters was increased during LPS treatment suggesting a regulatory role in ALI pathology. Interestingly, NFATc3^−/−^ mice showed increased bacterial infection in CLP mouse lungs due to decreased iNOS expression by macrophages, but significantly improved survival compared to WT mice when subjected to lethal CLP in presence of antibiotics. Although this could limit the clinical application of this approach, the combination of inhibiting the NFATc3 pathway and treatment with broad spectrum antibiotics seems to obviate this barrier and indicate that maintaining pulmonary barrier function is of paramount importance for survival and improved alveolar capillary gas exchange. Most importantly our data suggest that inhibition of NFATc3 and broad spectrum antibiotics could be used in combination in patients with severe sepsis. The mouse data has shown an impressive combined impact on survival that is not reported in earlier literature suggesting that blocking NFATc3 could be an effective adjunctive treatment for severe sepsis that would prevent the development of the acute respiratory distress syndrome (ARDS) and improve patient survival.

The cellular role of NFATc3-deficient macrophages in sepsis-induced ALI pathogenesis and pulmonary edema development is strongly supported by using novel high efficacy peptide inhibitors of NFATs in mouse lungs. GFP-VIVIT expression plasmids significantly reduced sepsis-induced pulmonary edema in CLP mouse models. This part of our study has certain limitations as GFP-VIVIT is delivered to all the structural cells in lungs and it is not specific for NFATc3. However, as other members of NFAT (NFATc1, NFATc2 and NFATc4) are not activated in macrophages during LPS stimulation and NFATc3 is not activated in lung PMVEC and alveolar type II epithelial cells, the observed decrease in sepsis-induced pulmonary edema can be attributed to macrophage specific NFATc3 activation. This approach was further supported by using a novel potent NFAT blocker CP9-ZIZIT that significantly attenuated sepsis-induced ALI and pulmonary edema. Administering the CP9-ZIZIT directly into the lung would be expected to have less systemic toxicities and greater efficacy in preventing loss of alveolar capillary barrier function compared to using other available delivery methods. Other researchers have shown that inhibition of NFATs with the cell permeable 11R-VIVIT peptide has protective effect in an experimental colitis model in mice [31], improved immunosuppression during fully mismatched islet allografts between BL/6 and C3H/HeN mouse strains [34], and attenuated both microgliosis and Amyloid β peptide (Aβ) plaque load in mouse models of Alzheimer's disease [35]. Also, treatment with 11R-VIVIT markedly attenuates albuminuria in diabetic db/db mice and alleviated mesangial matrix expansion and podocyte injury [36]. Furthermore, it was shown that blocking NFAT function was found to be beneficial in pulmonary arterial hypertension and acute pancreatitis [37, 38]. Role of NFAT members in different mouse disease models such as spontaneous pulmonary hypertension, diabetic retinopathy is well documented [39, 40]. In humans, liver allograft patients treated with Cyclosporine or Tacrolimus, showed increased NFAT dependent gene expression after 4–7 months or 8–12 months respectively [41]. In addition, the NFAT dependent gene expression was the maximum in all the subjects who rejected the transplant within one month indicating the critical role of NFAT's in inflammation and immunosuppression [41]. Recent studies also indicated NFAT dependent regulation of innate immune responses to fungal pathogens in rodents [42]. Thus, in addition to our data showing protection for ALI associated with severe sepsis, evidence that indicates the therapeutic potential of NFAT inhibition for a broad array of human maladies is accumulating.

Research efforts to identify NFAT inhibitors span over the last two decades but so far none of them performed well in the clinical trials [25, 26]. Interestingly, several alternative inhibitors including derivatives of cyclosporine like ISA247 were more potent and effectively inhibited Calcineurin, evaluated in phase III clinical trials against plaque psoriasis but did not result in to any commercial products so far [43, 44]. Natural compounds like gossypol [45], kaempferol [46] and Arctigenin [47] and other plant based compounds also showed variable degree of NFAT inhibition [48–50]. The plant based inhibitors may have less toxic side effects compared to the widely used immuno-suppressants, Cyclosporine and Tacrolimus but are less potent in terms of activity. Peptide inhibitors of NFATs are relatively less explored, less toxic and hold good potential as drug candidates. The newly developed cell permeable calcineurin inhibitor CP9-ZIZIT showed promising activity in attenuating LPS induced ALI and pulmonary edema in mouse models. We are currently improving the properties of CP9-ZIZIT by structure based docking studies to further increase binding affinity, cellular uptake and proteolytic stability. In summary, our study supports the conclusion that NFATc3 has a pivotal role in mediating sepsis-induced ALI pathogenesis and pulmonary edema. Studies are underway to generate higher efficacy derivatives of CP9-ZIZIT and evaluate them in mouse post sepsis-ALI disease models.

## MATERIALS AND METHODS

### Cells

Bone marrow cells were differentiated into mature macrophages (BMDMs) from wild type C57BL/6 (WT) and NFATc3^−/−^ mice in DMEM supplemented with 10% FBS, 1% penicillin/streptomycin and 20 ng/mL recombinant mouse M-CSF added on Day 1, 3 and 5. Cells were differentiated up to 7 days in total [4–6]. Mouse lung total macrophages were isolated by collagenase digestion, purified by adherence for 1 hour, plated in DMEM supplemented with 10% FBS, 1% penicillin/streptomycin [51]. Mouse PMVEC were isolated by Miltenyi CD31 magnetic beads. AEC type II cells were isolated as described earlier [52].

### Mice

NFATc3^−/−^ in C57BL/6 background and wild type C57BL/6 mice were purchased from Jackson Laboratories and breeding colonies were maintained at the Ohio State University, Columbus. All the animal experiments and procedures were conducted with protocols approved by the Institutional Animal Care and Use Committee (IACUC) of OSU.

### Antibodies and fine chemicals

Recombinant mouse M-CSF was purchased from PeproTech (Catalog# 315-02, Rocky Hill, NJ). Antibodies for immunoblotting of proteins were purchased from different companies as indicated: NFATc3, NFATc2, NFATc1-Cell Signaling Technology (Catalog# 4998, 4389, 8032, Danvers, MA), NFATc4–Santa Cruz (sc-13036, Dallas, TX), and α-Tubulin–Santa Cruz (sc-8035). All the antibodies were validated in our earlier publications [6]. All primers and oligonucleotides were synthesized by IDT (Coralville, IA) and listed in supplement I. ChIP One Day Chromatin Immunoprecipitation kit was purchased from QIAGEN (Catalog# 334471, Germantown, MD).

### Vectors and transfection reagents

Myc-DDK tagged human NFATc3 expression vector (catalog# RC212523) was purchased from Origene (Rockville, MD). Transient transfections were carried out using cell type-specific Lonza Electroporation kit using Amaxa Electroporator. GFP and GFP-VIVIT expression plasmids were obtained from Addgene (Cambridge, MA) and liposomes were prepared according to our published protocol [2, 4].

### Western blot analysis

BMDMs or lung interstitial macrophages from WT or NFATc3^−/−^ mice were harvested, and cell extracts were prepared in 1X RIPA buffer (Cell signaling Technologies catalog# 9806) supplemented with protease inhibitors. An equal amount of protein was analyzed by Western blotting for the protein of interest using its specific antibodies according to standard protocols.

### Chromatin immunoprecipitation assay

Binding of NFATc3 protein to NFATc3 consensus sequence in the TNFα promoter was assayed using QIAGEN Epitect Oneday Chromatin Immunoprecipitation kit as described earlier [53].

### Mouse pulmonary microvascular endothelial cell (PMVEC) monolayer permeability

Millicell hanging cell culture inserts (3.0 μm pore size) were seeded with mouse PMVEC isolated by CD31 Miltenyi magnetic micro beads up to 100% confluence and allowed to grow for 16 hours. NFATc3^−/−^ or WT BMDM grown in 24 well plates were stimulated with LPS for 8 hrs. The cell culture inserts containing PMVEC monolayer were transferred to 24 well plates containing LPS stimulated macrophages and co-cultured for an additional 24 hrs. FITC-Dextran (mol wt 10,000) was added to the upper compartment of the PMVEC monolayer inserts at the time of initiating the co-culture. Increased permeability of the EC monolayer was assessed by the flux of FITC-Dextran across the EC monolayers into the lower compartment of the wells by measuring fluorescence of the culture medium at 492 nm excitation and 523 nm emissions.

### Mouse models of sepsis

Cecal ligation puncture (CLP) sepsis models were established according to Cuenca *et al.* [54]. In an anesthetized mouse, the cecum is exposed and ligated below ileo-cecal valve and two perforations were made across the cecum using 21G needle. The cecum is then compressed to express minute amount of fecal material. To establish moderate CLP sepsis the cecum was ligated at less than 1.0 cm from the distal end and for severe sepsis about 2.0 cm of the cecum from distal end was ligated. Pulmonary edema, arterial oxygenation, and inflammation were evaluated in moderate CLP sepsis models. Survival studies were carried out in the severely septic CLP mice. For the sham surgery group, the cecum was exposed and repositioned back in the abdominal cavity before suturing the skin layers separately. LPS (15 mg/kg) or the same volume of normal saline was administered into intraperitoneal cavity of WT or NFATc3^−/−^ mice and arterial oxygenation was measured by pulse oximetry.

### Pulmonary arterial oxygenation by pulse oximetry

Following LPS injection, the fur around the neck of each mouse was shaved and pulmonary arterial oxygenation status of each mouse was measured at 8 and 24 hours by MouseOx Plus (Starr Life Sciences Corp, USA) in accordance with manufacturer's instructions. Each mouse was allowed to acclimatize for 1 hour after placement of a collar clip sensor. Oxygen saturation measurements were recorded for 5 minutes for 5 times and then averaged.

### Pulmonary neutrophilic inflammation and edema

WT and NFATc3^−/−^ mice were subjected to moderate CLP and after 24 h post sepsis, cytospin slides were prepared from broncho alveolar lavage fluid (BALF). Cytospin slides were stained using Hema 3.0 and neutrophils and macrophages were counted and differences were compared for statistical significance. The protein content in cell-free BALF was determined as described earlier [4].

### Lung capillary leakage and wet/dry ratio measurement

Evans Blue–labeled albumin (EBA) was administered intravenously in Sham/PBS and CLP/LPS group of mice. After 30 minutes post EBA injection, the mice were euthanized to assess vascular leakage in the right lobes. Additionally, the left lobe of the lung was excised, weighed, and dried in the oven at 60°C for 18 hours. EBA extravasation and the difference between the lung wet/dry ratios were quantitated in sham and CLP mice as described earlier [4].

### Kaplan meier survival curves

NFATc3^−/−^ and WT mice were subjected to severe sepsis by CLP and observed for number of deaths over a period of 96 h. Kaplan Meier survival curves of different mice groups was plotted using OriginProâ statistical software [55].

### Adoptive transfer of Macrophages

Mouse lung resident macrophages from recipient mice (*n* = 5) were depleted by nasal insufflation of 50 μL of clodrosome or control liposomes (Encapsula LLC). After 72 h of clodrosome delivery, 1 × 10^6^ lung macrophages (isolated by collagenase/DNase digestion, adherence purified) from donor mice are delivered in to trachea of the recipient mice. Mice transferred with WT to WT, KO to KO (NFATc3^−/−^ to NFATc3^−/−^, WT to NFATc3^−/−^ or NFATc3^−/−^ to WT macrophages are subjected to LPS or PBS (i.p) challenge. BALF protein, cytokines and lung wet/dry ratio were determined in the recipient mice subjected to LPS/PBS treatments.

### Statistical analysis

All the experiments were repeated three to four times to test for consistency and representative data sets are presented for EMSA, WB and flow cytometry. For all the data sets involving *in vitro* cell assays and mouse CLP/LPS sepsis models, statistical analyses were performed using non-paired Student's *t* test or ANOVA and *p* ≤ 0.05 was considered significant. Error bars represent standard deviation/standard error. Statistical significance is indicated in corresponding figure legends.

## SUPPLEMENTARY MATERIALS FIGURES AND TABLE


