# Supplementary material for: Inhibition of nuclear factor of activated T cells (NFAT) c3 activation attenuates acute lung injury and pulmonary edema in murine models of sepsis
Source: Oncotarget. 2018 Jan 25;9(12):10606–20. doi: 10.18632/oncotarget.24320 (PMC5828182; doi:10.18632/oncotarget.24320)
Supplement: Supplementary file 1 [file oncotarget-09-10606-s001.pdf]

# Inhibition of nuclear factor of activated T cells (NFAT) c3 activation attenuates acute lung injury and pulmonary edema in murine models of sepsis

## SUPPLEMENTARY MATERIALS

| Sample ID | Sequence |
|---|---|
| CP9-ZIZIT | cyclo(f•RrRrQ)-PEG$_2$-GPHPZIZITGPHEEK-NH$_2$ |
| CP9-VAVAA | cyclo(f•RrRrQ)-PEG$_2$-GPHPVAVAAGPHEEK-NH$_2$ |

f = D-phenylalanine, • = L-2-naphthylalanine, Z = L-tert-leucine

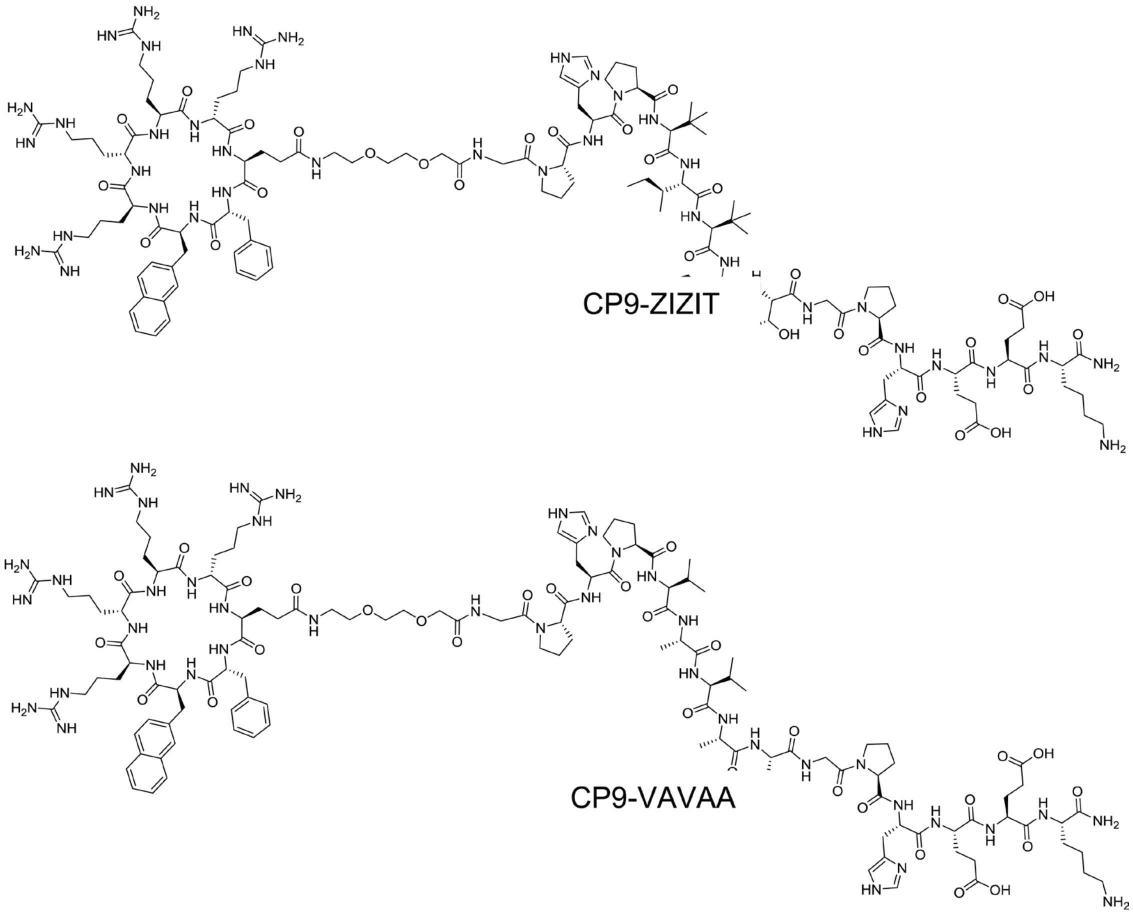


**Supplementary Figure 1: Molecular sequence and structures of CP9-VAVAA, CP9-ZIZIT.**

| Sample ID | Sequence |
|---|---|
| CP9-ZIZIT-Biotin | cyclo(f•RrRrQ)-$PEG_2$-GPHPZIZITGPHEEK($PEG_4$-Biotin)-$NH_2$ |
| CP9-VAVAA-Biotin | cyclo(f•RrRrQ)-$PEG_2$-GPHPVAVAAGPHEEK($PEG_4$-Biotin)-$NH_2$ |

f = D-phenylalanine, • = L-2-naphthylalanine, Z = L-tert-leucine

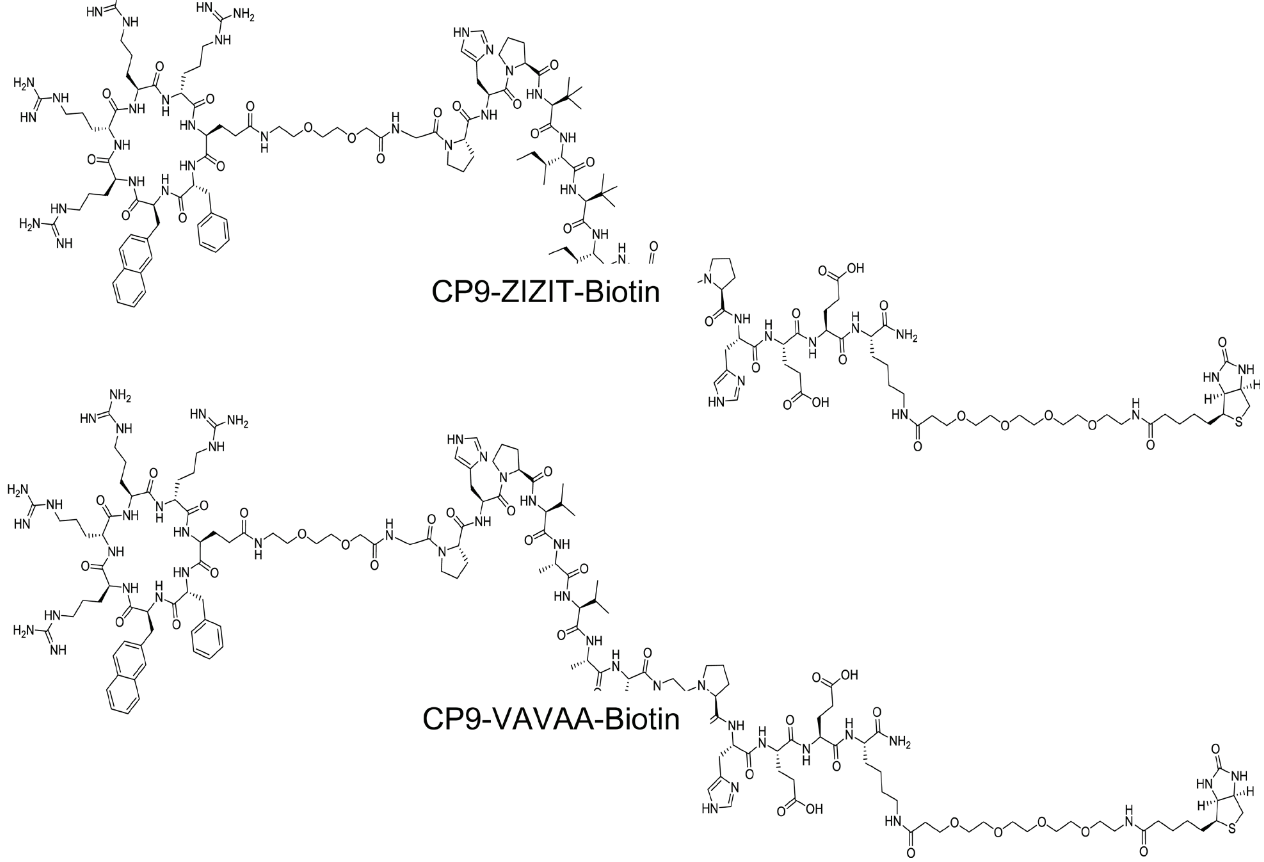


**Supplementary Figure 2: Molecular sequence and structures of CP9-VAVAA-Biotin, CP9-ZIZIT-Biotin.**

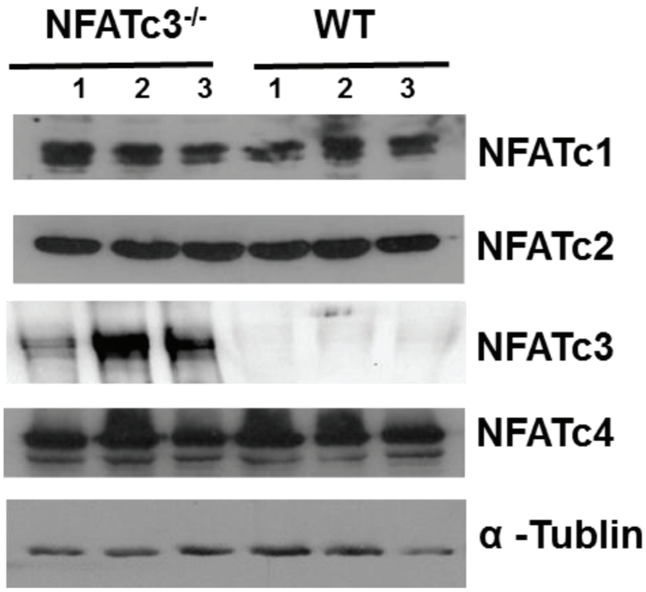


**Primer sequences and Oligo's:**

TNFα promoter ChIP qPCR forward primer: 5' TTATGCACCCAGCTTTCAG 3'

TNFα promoter ChIP qPCR reverse primer: 5'CAATCAGGAGGGTGTGTGTG 3'

**Mouse CCR2 promoter oligo used for electrophoretic mobility shift assay:**

5' Biotin-CC<u>AGGA AA</u>TGCCAAGGATGTTA<u>AGGAAA</u>TG GTTGC 3'

**Supplementary Figure 3: Expression levels of NFATc1, NFATc2, NFATc3 and NFATc4: Total cell lysates from lung tissues of NFATc3$^{-/-}$ and WT mice (*n* = 3) were analyzed by immunoblotting using NFATc1, NFATc2 and NFATc4 specific antibodies.** NFATc3$^{-/-}$ mice show absence of NFATc3 protein expression where as other NFAT's are expressed normally.

**Supplementary Table 1: LPS stimulated NFATc3$^{-/-}$ macrophages show attenuated expression of inflammatory genes**

| S. No. | Gene | Fold Up-regulation | Fold Down regulation |
|---|---|---|---|
| 1. | TNFα | | –3.06 |
| 2. | iNOS | | –4.1 |
| 3. | CCL2 | | –4.56 |
| 4. | CCR2 | | –3.96 |
| 5. | IL22 | +6.72 | |
| 6. | TLR5 | +9.04 | |
| 7. | LTa | +4.16 | |
| 8. | Kng1 | +4.24 | |
| 9. | IL9 | +9.22 | |

Pooled BMDM from WT and NFATc3$^{-/-}$ mice ($n$ = 4) were treated with LPS (100 ng/mL for 8 h) and total RNA was isolated using RNeasy plus, genomic DNA was removed by DNA digestion columns. Resulting total RNA was reverse transcribed and analyzed using SABioscience PCR array (PAMM-077) for inflammatory genes. Highly upregulated and downregulated genes are shown in the table.
